# Epizootic reptilian ferlavirus infection in individual and multiple snake colonies with additional evidence of the virus in the male genital tract

**DOI:** 10.1038/s41598-021-92156-5

**Published:** 2021-06-16

**Authors:** Chutchai Piewbang, Sabrina Wahyu Wardhani, Panida Poonsin, Jakarwan Yostawonkul, Poowadon Chai-in, Sitthichok Lacharoje, Thanyarat Saengdet, Taksa Vasaruchapong, Suwimon Boonrungsiman, Piyaporn Kongmakee, Wijit Banlunara, Anudep Rungsipipat, Tanit Kasantikul, Somporn Techangamsuwan

**Affiliations:** 1 Department of Pathology, Faculty of Veterinary Science, Chulalongkorn University, Bangkok, 10330 Thailand; 2 Animal Virome and Diagnostic Development Research Group, Faculty of Veterinary Science, Chulalongkorn University, Bangkok, 10330 Thailand; 3 The International Graduate Course of Veterinary Science and Technology (VST), Faculty of Veterinary Science, Chulalongkorn University, Bangkok, 10330 Thailand; 4 National Nanotechnology Center (NANOTEC), National Science and Technology Development Agency (NSTDA), Thailand Science Park, Pathumthani, 12120 Thailand; 5 Siam Serpentarium, Siam Park Recreation Co., Ltd., Bangkok, 10520 Thailand; 6 Snake Farm, Queen Saovabha Memorial Institute, The Thai Red Cross Society, Bangkok, 10330 Thailand; 7 The Zoological Park Organization under The Royal Patronage of H.M. The King, Bangkok, 10800 Thailand; 8 Clemson Veterinary Diagnostic Center, Clemson University, Columbia, SC 29229 USA

**Keywords:** Infectious-disease diagnostics, Pathogens, Virology, Infection

## Abstract

Reptilian ferlavirus, a pathogen of serious concern in snakes, has been reported in Western countries, but little is known about its prevalence in Thailand, where many snake breeding farms are located. In this study, we investigated the reptilian ferlavirus via swab samples derived from 49 diseased snakes and 77 healthy snakes as well as tissue samples taken from nine dead snakes from five independent snake farms. Using molecular detection, we found the ferlavirus in 8.16% of diseased snakes, but not in healthy snakes. Out of nine farmed snakes, eight snakes derived from four farms were found to be positive. Four complete genome sequences of the ferlavirus were successfully obtained and phylogenetically clustered to the highly pathogenic ferlavirus. Tissue tropism of the ferlavirus was identified in various epithelial cell types using the in situ hybridization technique. Interestingly, the hybridization signals were strongly labeled in the male genital tract. Transmission electron microscopy was used to support the ferlaviral localization in the male genital tract. This study provides the first evidence of ferlavirus localization in the male genital tract and contributes to the knowledge about ferlavirus epidemiology, indicating that there needs to be further awareness and elucidation regarding vertical transmission of reptilian ferlavirus.

## Introduction

Reptilian ferlaviruses are serious pathogens that cause respiratory and neurological diseases in various species of snakes and reptiles^1^. Ferlavirus was first discovered in snakes showing respiratory disease in Switzerland in 1972, and it was tentatively named the Fer-de-lance virus (FDLV)^2^. Since then, the virus has been identified in various snake species, while related FDLVs have been sporadically documented in a variety of reptiles, resulting in what was formerly described as the ophidian paramyxovirus (OPMV)^3^. To date, there is only a *reptilian ferlavirus* species, accommodating the genus *Ferlavirus*, family *Paramyxoviridae*^4^. Various snake families are susceptible to ferlavirus infection, including colubrids, crostalids, elapids, boids, and pythonids, and the clinical severity of infection varies depending on the infected snake species^5^. Experimental infection of ferlavirus in various snakes confirmed that respiratory disease is the most common clinical presentation, which supports clinical features described in natural infections of many captive snake species^3,6^. Clinical severity of the infected snakes is increased when the animals are opportunistically infected by either bacteria or other viruses, such as retrovirus and adenovirus, which could lead to severe inflammation and thus become fatal^6–8^.

Apart from pulmonary lesions, neurological signs are a well-recognized clinical symptom in ferlavirus-infected snakes. Non-suppurative meningoencephalitis and demyelination were pathologically described in ferlaviral-infected cases^9,10^. Regarding ferlavirus localization in infected snakes, viral tropism in the lung, liver, kidney, and brain has been reported, while viral RNA was diffused in various organs, including the pancreas, intestine, lung, liver, kidney, and brain^3,10–14^. This leads to questions as to whether the ferlavirus may cause viremia and disseminate to other organs^15^.

Evidence of ferlaviral localization in lungs and identification of viral genomes in respiratory discharge has focused more attention on virus transmission. A previous study found that ferlavirus transmission is very contagious through both direct contact and aerosols^16^. Identification of the ferlaviral genome in the oral and cloacal excretion of infected snakes emphasized that excretions may serve as potential infective substances^7,17^. To the best of our knowledge, there are no reports regarding the vertical transmission of ferlavirus, as previously indicated^16^, The role of ferlavirus transmission needs to be further investigated to better understand disease prevention and management.

The live animal trade and the import of fresh raw meat may introduce carry-over pathogens into native species, serving as a potential source of disease outbreak and economic loss. The countries of Southeast Asia (SEA) have been recognized as centers for snake farming for various purposes over the last 20 years^18,19^. Thus, there will be a significant economic impact on snake trading, which will result in substantial losses, if an outbreak of ferlavirus infection occurs. Although ferlavirus infection in snakes is of grave concern in Europe and the Americas^20^, information regarding ferlavirus infection in snakes from Thailand, where over 100 snake breeding farms are located, is lacking^18,21–23^. Epidemiology and disease surveillance of ferlavirus infection in all captive, farmed, and pet-owned snakes in Thailand are needed. Here, we have investigated the reptilian ferlavirus infection in non-captive snakes and those from snake breeding farms in Thailand using reverse transcription-polymerase chain reaction (RT-PCR). Viral localization in various tissues was then confirmed using in situ hybridization (ISH). Evidence of ferlaviral genomes and their gene products were found in epithelial cell linings of the efferent duct and epididymis of infected snakes, which suggests the possible role of sexual and, thus, vertical transmission of ferlavirus infection. Other potential viruses associated with inclusion body diseases, including arenaviruses and retroviruses, were also tested.

## Results

### RT-PCR detection of snake ferlavirus in individuals and breeding farms

Testing of oral and cloacal swabs derived from diseased and healthy individual snakes revealed similar results in both oral and cloacal swabs. RT-PCR-positive results were found in 8.16% of diseased (4/49) and 0% of healthy (0/77) snakes. Ferlavirus was detected in snakes from multiple breeding farms (i.e., farms A, B, D, and E, while the samples from farm C tested negative). Among the tested groups, the ferlaviral genome was detected in various fresh tissues, including lung, liver, kidney, spleen, and pooled male genital tracts of diseased snakes (Table 1). Note that the lung (4/5) of necropsied snakes was the organ where the virus was most often detected. Interestingly, pooled male genital organs were positive for ferlavirus RT-PCR in both tested farms. In addition, there were no available fresh spleen samples of two snakes in colonies C and D, resulting in no results from RT-PCR testing on those samples. Moreover, reovirus, arenavirus, and retrovirus were not detected in any fresh tissue and swab samples.

**Table 1:** Summarized sample information and testing results to identify the reptilian ferlavirus.

Sample source	Diseased snakes/total snakes	Snakes	Tissue	RT-PCR	ISH	TEM	Accession no
Colony A	2/2	Big-eyed pit viper no. 1	Liver	–	N/A		
			Kidney	+	–		
			Male genital tract	+	+	+	MW976960
			Intestine	+	+		
		Big-eyed viper no. 2	Kidney	+	N/A		
			Liver	+	N/A		
			Intestine	+	+		
Colony B	6/7	Ball python no. 1	Lung	+	+		MW976761
			Liver	–	–		
			Kidney	–	–		
			Spleen	+	N/A		
		Ball python no. 2	Lung	+	+		
			Liver	–	–		
			Kidney	+	–		
			Spleen	–	N/A		
Colony C	6/7	Ball python	Lung	–	–		
			Liver	–	–		
			Kidney	–	–		
			Spleen	N/A	–		
Colony D	5/5	Corn snake	Liver	+	–		MW976962
			Kidney	+	–		
			Spleen	N/A	–		
Colony E	4/6	Cobra no. 1	Lung	+	+		
			Liver	–	–		
			Kidney	–	–		
			Male genital tract	+	+	+	MW976963
		Cobra no. 2	Lung	+	N/A		
			Liver	–	–		
			Kidney	–	N/A		
		Cobra no. 3	Liver	+	–		
			Kidney	+	–		
			Spleen	+	N/A		

RT-PCR: Reverse transcription polymerase chain reaction; ISH: In situ hybridization; TEM: Transmission electron microscopy; +: Positive; –: Negative; N/A: Not tested due to lack of available samples.

### Whole genome and phylogenetic analysis of obtained ferlavirus

**Figure 1:**
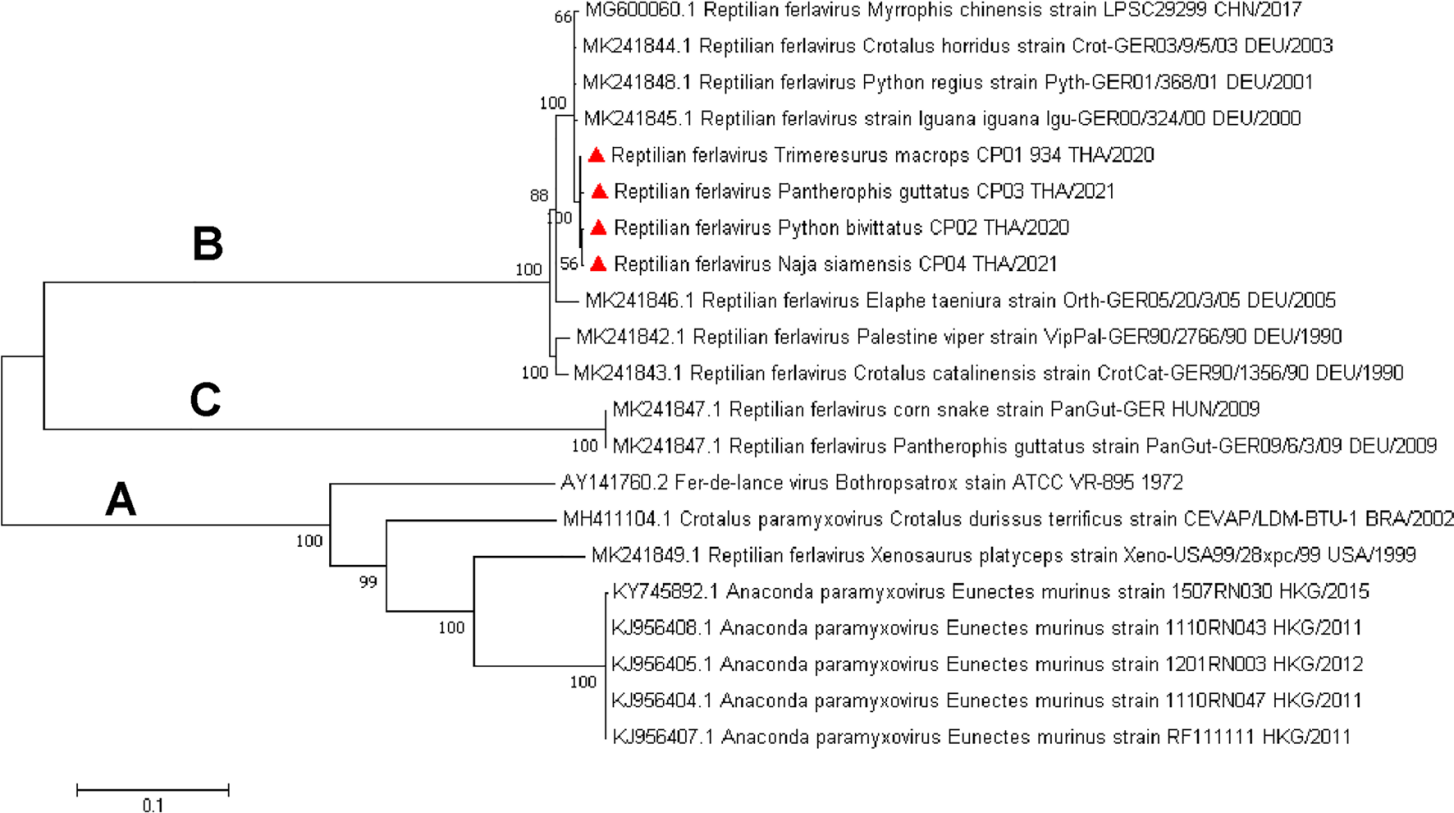
Phylogenetic analysis based on complete coding sequences of reptilian ferlaviruses. The ML topology was constructed using the GTR + I + G model and 1000 bootstrap replicates. Bootstrap values (%) are indicated above node even > 50%. Reference genome sequences studied in this analysis were labeled with their respective GenBank accession numbers. The reptilian ferlavirus phylogenetic tree showed three different phylogenetic clades (clades A–C) with high bootstrap support (100%). The obtained reptilian ferlavirus strains detected in this study are indicated by a red triangle and are clustered in clade B.

As a result of our initial detection and characterization of a portion of the L gene of ferlavirus and due to the fact that we have limited RNA samples, we selected four extracted RNA samples from the two pooled male genital tracts of snakes in colonies A and E, one lung sample from ball python no. 1 of colony B, and a liver sample of a necropsied snake from colony D to obtain the whole ferlavirus genome. Using multiple primer sets and Sanger sequencing, we successfully amplified the whole genome of four ferlavirus strains, tentatively naming them snake ferlavirus strains CP01-934 TH/2020, CP02 TH/2020, CP03 TH/2021, and CP04 TH/2021 (derived from colonies A, B, D, and E, respectively). The complete coding sequence of obtained ferlavirus sequences was submitted to the GenBank database as accession nos. MW976960–ME976963. Genetic diversity among the four obtained snake ferlaviruses was low. When they were genetically divergent from previously published ferlaviruses, genetic diversity was 1–2.29%. Phylogenetic analysis based on complete coding sequences showed that the reptilian ferlavirus sequences were separated into three genetic distinctions (A–C). The ferlavirus sequences obtained in this study were clustered in group B and formed a sub-branch within the German and Chinese reptilian ferlavirus isolates (Fig. 1).

### Histology and in situ hybridization

**Figure 2:**
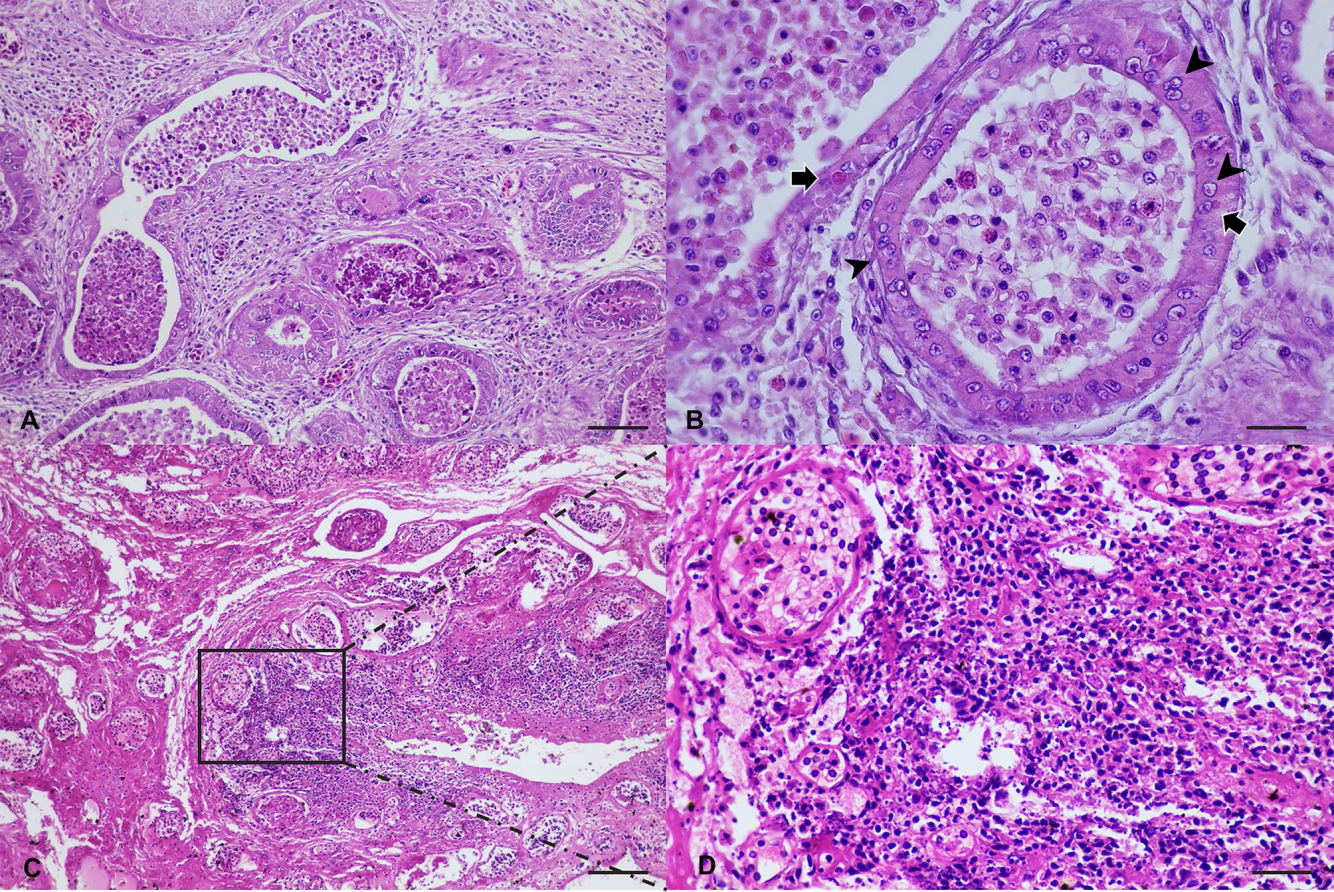
Histopathological examination of the snake positive for reptilian ferlavirus infection. Epididymis (**A**,**B**) and intestine (**C**,**D**) of a big-eyed pit viper from colony A. (**A**) Locally extensive to diffuse necrotizing to mixed vasitis. (**B**). Tubular epithelial cells of epididymis contain 2–3 μm eosinophilic intranuclear inclusion bodies (arrowheads) and fewer similar eosinophilic inclusion bodies, as noted in the cytoplasm (arrows). (**C**) Marked, locally extensive, segmental, full thickness, necrotizing enteritis. (**D**) Higher magnification reveals severe necrosis of intestinal epithelium with fibrin clumping. H&E. Bars indicate 850 μm for (**A**) and (**C**) and 170 μm for (**B**) and (**D**).

Regarding available formalin-fixed paraffin-embedded (FFPE) sections, histological examination was limited to cases that tested positive via ferlavirus RT-PCR testing, and the histological findings were described based on available tissue. Histological lesions of ferlavirus-positive snakes were consistent, represented by severe necrosis in several organs. Histological findings were detailed according to the most significant changes among the cases, and more histological details were described collectively in some specific cases. For the lung, severe inflammatory reaction was evidenced in all cases characterized by severe lymphoplasmacytic infiltration of the pulmonary interstitium with massive fibrin and necrotic tissue precipitation in the faveolar spaces. Less severe lesions were noted in snakes from colony B compared with those from colony E. For liver sections, normal architecture was disrupted, and many hepatocytes diffusely formed irregular lobules or were dissociated. Sections from the male reproductive tract (epididymis) derived from snakes in colonies A and E contained variably ectatic acinar/tubular structures surrounded by loosely edematous stroma that was multifocally obscured by variably dense dissecting interstitial and perivascular infiltrates of mixed inflammatory cells, including predominant plasma cells, lymphocytes, heterophils, and small areas of hemorrhage (Fig. 2A). Lumens of several glands were filled with variable pools of necrotic debris intermingled with foamy histiocytes, heterophils, and rare multinucleated cells. Acinar/tubular epithelial cells were variably segmentally lost or attenuated, and occasional epithelial cells contained eosinophilic intranuclear inclusion bodies that were 2–3 μm in diameter. Fewer similar eosinophilic inclusion bodies were noted in the cytoplasm (Fig. 2B). Changes in sections from the gastrointestinal tract of all examined cases were similar, while those from colony E were more severe. These sections were entirely hypereosinophilic with extensive loss of cellular detail but maintained some degree of tissue architecture (Fig. 2C). The superficial mucosa was blunt and multifocally lost, and the exposed stroma was covered by abundant hemorrhage and fibrin intermixed with clumps of rod-shaped bacilli and coccoid bacteria. The submucosal layer was markedly expanded via edema, pools of extravasated erythrocytes, and fibrin accumulation (Fig. 2D). Lumens of occasional vessels were obliterated by variable aggregates of organizing mats of eosinophilic acellular fibrillar material suggestive of fibrin thrombi. The walls of such vessels were often rimmed by dense aggregates of similar fibrillar material. For kidney sections, there were areas of renal tubular loss with associated dissecting bands of dense interstitial fibrosis that multifocally surrounded variably ectatic renal tubules. Rare renal tubules were necrotic and contained aggregates of eosinophilic cellular and nuclear debris.

**Figure 3:**
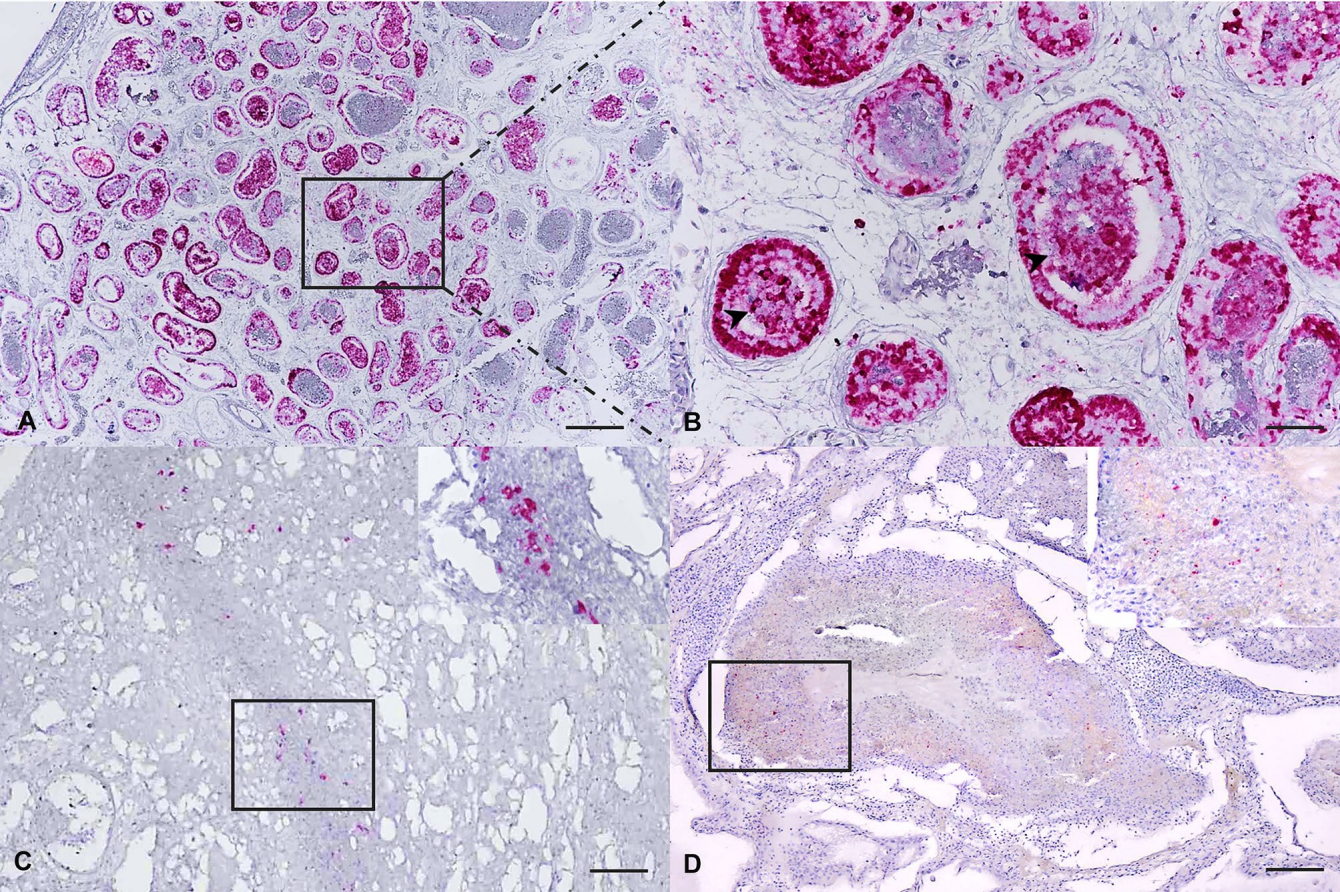
Reptilian ferlavirus RNA in the snake tissues. Epididymis (**A**,**B**) and intestine (**C**) of a big-eyed pit viper from colony A and Lung (**D**) of a cobra from colony E. (**A**) Diffuse, strong hybridization signals (red precipitates) in most of the epididymis and efferent ducts. (**B**) Strong nuclear and cytoplasmic signals (red precipitates) are localized in the tubular epithelia and in free-floating cells (arrowheads) in epididymal ducts. (**C**) Cytoplasmic hybridization signals (red precipitates) are labeled in submucosal epithelial cells (inset). (**D**) Rare hybridization signals (inset) are seen in sloughed and necrotic cells in the faveloar space. ISH for reptilian ferlavirus. Bars indicate 45 μm for (**A**,**C**–**D**) and 120 μm for (**B**).

The ISH technique was applied in all available FFPE sections in order to validate RT-PCR testing results and to demonstrate the virus’s presence in various tissues, where it presented with pathological changes. Several tissues, including the lung, intestine, and the male genital tract, revealed positive hybridization signals (Fig. 3). The signals were localized in most epithelia and/or the sloughed epithelia of bronchi and submucosal areas of infected snakes. Interestingly, the cytoplasmic hybridization signals of ferlaviral genomes were abundant in the tubular epithelium of the epididymis and in the efferent duct. No immunolabelling signals presented in the negative controls (Supplementary Fig. S1). Both nuclear and cytoplasmic hybridization signals were also detected in various cells in the tubules. Summary details of ISH testing are described in Table 1.

### Ultrastructural demonstration of reptilian ferlavirus

**Figure 4:**
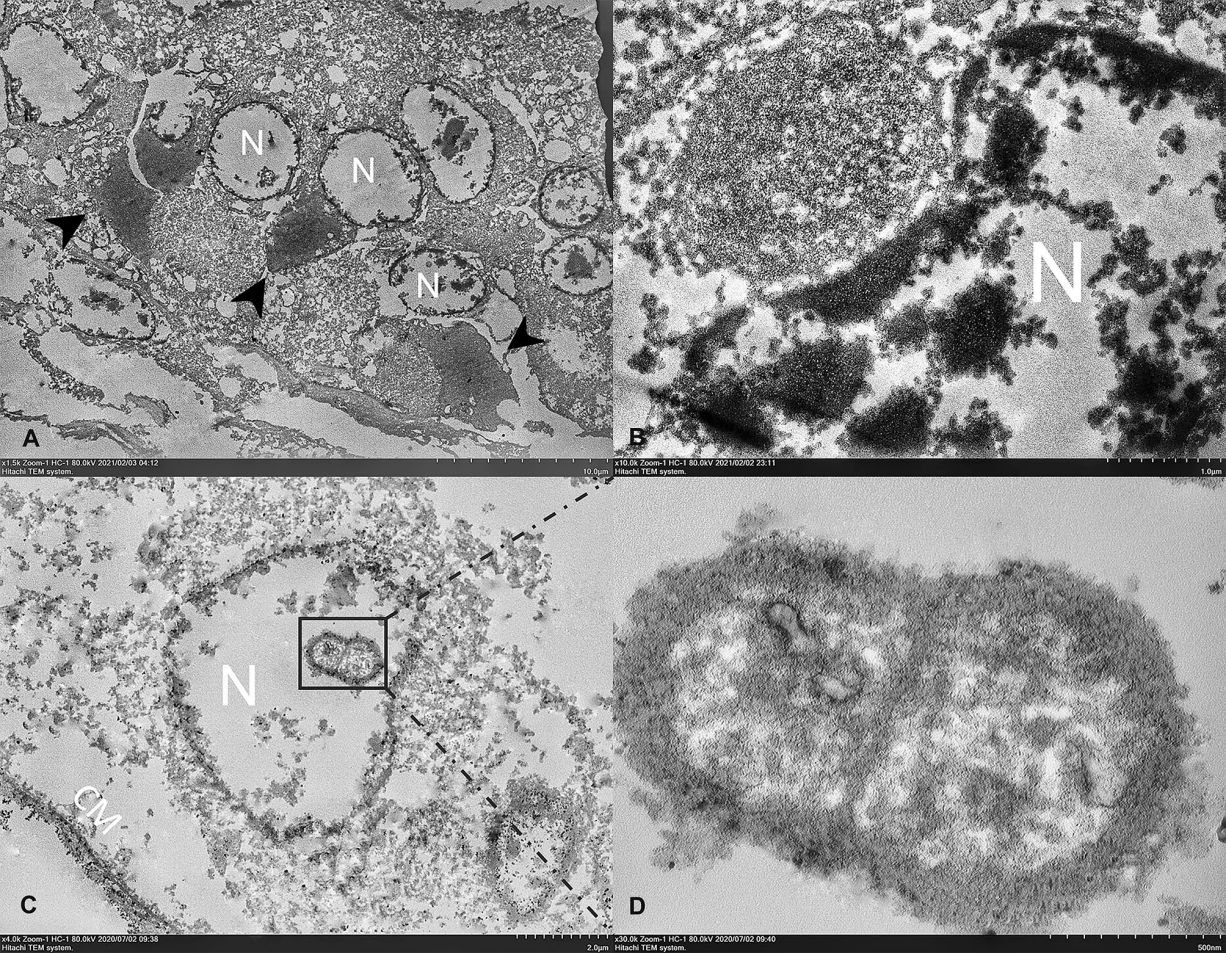
Transmission electron microscopic images of reptilian ferlavirus in the snake epididymis. Representative TEM images from a big-eyed pit viper snake from colony A. (**A**). Intracytoplasmic, large, electron-dense viral factories (arrowheads) were observed in multiple degenerated epithelial cells represented by cellular vacuolation and a disrupted nuclear membrane (N). (**B**) A cytoplasmic inclusion body contains numerous pleomorphic, electron-dense viral nucleocapsid particles displacing the nucleus (N). (**C**) Ferlaviral ribonucleocapsid particle was seen in the nucleus (N). (**D**) Pleomorphic ribonuleocapsid with herringbone-like structure. Bars indicate as described in figures. *CM* cellular membrane.

As evidence of strong ISH signals were observed in the male reproductive tract and to provide additional support for our ISH results, transmission electron microscopy (TEM) was used to ultrastructurally demonstrate the ferlaviral particles in the male reproductive tract (Fig. 4). Overall, the observed cells in the epididymis of both snakes (big-eyed pit viper No. 1 from colony A and cobra No. 1 from colony E) revealed severe degeneration and necrosis, represented by cellular vacuolation and disrupted epithelial membrane (Fig. 4A). The interpretation of those tissues was cofounded by degenerative changes. Numerous densely aggregated intracytoplasmic electron-dense materials were found frequently in the tubular basement membrane of the epididymis and efferent duct (Fig. 4B). Electron-dense nucleocapsid particles that packed and eccentrically replaced the round-to-oval flattened nucleus were seen in the freely floating cells in the efferent tubules and in the lumen of the epididymis (Fig. 4C). The materials contained numerous pleomorphic nucleocapsid filaments with estimated sizes ranging from 200–400 nm. Freely floating virions were seen in the vas deferens of both examined snake tissues (Fig. 4D).

## Discussion

Reptilian ferlavirus is an important, serious, and contagious pathogen in snakes in both individual and farmed specimens^5,20,24^. Reptilian ferlavirus infection in snakes has been reported worldwide on multiple continents; however, information regarding the infection in Asia, especially Thailand (one of the Southeast Asian countries that exports the most snake products), is limited. In this study, we investigated the reptilian ferlavirus infection in both diseased and healthy individuals, and for the first time, in snake breeding farms in Thailand. The prevalence of ferlavirus infection in the individual animals was low. Only diseased snakes and unhealthy animals were found to be positive for ferlavirus. It is notable that the number of infected snakes was higher in animals derived from breeding farms. These findings may support evidence of a low prevalence of reptilian ferlavirus infection in individual snakes in Thailand; however, the infection rate is higher in animals with close contact in population-dense environments (e.g., breeding farms), which is in agreement with previous studies that indicate that the virus is pathogenic and very contagious to neighbors^24,25^. However, interpretation of epidemiology results from this study should be done cautiously since different sample sources (i.e., swab samples and homogenized fresh tissues) between individual and farmed snakes were utilized. Several studies have indicated that fresh tissue samples are more useful for virus detection than swab samples^25,26^. Our low number of tested animals may also have affected the results and their interpretation.

Phylogenetic analysis based on full-length ferlavirus sequences detected in this study revealed genetic homology, and they were clustered with a previously indicated pathogenic lineage^8,25^. This finding suggests that this pathogenic ferlavirus was circulating in the Thai snake population. However, since we could not detect the other lower pathogenic ferlavirus lineages in the tested snake, a definitive finding of ferlavirus infection presenting abnormal clinical signs is warranted. Also, our study lacked evidence regarding immunosuppression leading to secondary bacterial infection and contributing to the severity of ferlavirus in the infected snakes^25^. Further, studies on a larger scale and investigation of bacterial coinfection should be done to reach a definitive conclusion.

In the collection of necropsied snakes derived from independent breeding farms in Thailand, we found four out of five farms were positive for ferlavirus, suggesting the epizootic nature of the disease. Although this study was investigatory and not limited to dead snakes, our data support the premise that this virus may serve as the associated cause since we found no other viral pathogens by showing ferlavirus genome localization in tissues using the ISH. Ferlavirus-positive RT-PCR results were most prevalent in lung samples, while kidney and liver samples tested positive, supporting a previous study that indicated that lung, liver, and kidney were more reliable tissues when screening for the presence of ferlavirus^8^. Despite finding positive RT-PCR results for ferlavirus in several organs, ISH signals were found in only a limited number of organs, including lung, intestine, and male reproductive organs. This discrepancy in results may be explained by several factors, including hematogenous spreading and/or different sensitivities among the tests.

Importantly, we confirmed ferlavirus infection in multiple snake breeding farms using ISH, and our results revealed that ferlavirus is not limited to the lung, but it is also present in male reproductive organs. This finding provides novel information regarding the localization of this virus in the male reproductive tract. The presence of hybridization signals prompted us to further illustrate the virus in the male reproductive tract using TEM, which confirmed that the viral particles exist in the epithelia of the epididymis and efferent duct, and in freely floating cells in the lumens of the epididymis duct and efferent duct. This finding may be explained via vertical transmission of the reptilian ferlavirus, but further explorations are needed to confirm this hypothesis.

To date, sperm collection and artificial insemination of snakes are useful and important not only for threatened snake conservation, but also to call attention to disease control^27–29^. Based on the results obtained in our study, we identified the presence of ferlavirus in the male reproductive tissues for the first time, and we speculate that the male reproductive discharge (such as semen) of infected snakes may serve as a potential source of infection. Thus, screening of ferlavirus in collected semen after collection and before artificial insemination is essential.

In conclusion, this study identified reptilian ferlavirus in both individual snakes and snakes from multiple snake breeding farms in Thailand. Whole genome characterization and phylogenetic analysis support the fact that pathogenic reptilian ferlavirus lineage is circulating in Thailand. In addition, tissue localization of reptilian ferlavirus was evidenced in lung, intestine, and male reproductive organs, confirming the infection of this virus. Ferlaviral localization in male reproductive organs was definitively supported by ultrastructural investigation using TEM. Vertical transmission of reptilian ferlavirus needs further elucidation in future studies.

## Materials and methods

### Animals and samples

From 2019 to 2021, five independent snake breeding colonies suffered outbreaks of an unknown clinical disease resulting in high mortality rates: colony A (a colony of the big-eyed pit viper, *Trimeresurus macrops*, *n* = 2, 100% mortality rate [2/2]), colonies B & C (2 colonies of the ball python, *Python bivittatus*, *n* = 7, 85.71% mortality rate [6/7]), colony D (1 colony of the corn snake, *Pantherophis guttatus*, *n* = 5, 100% mortality rate [5/5]), and colony E (1 colony of the cobra, *Naja siamensis*, *n* = 6, 66.67% mortality rate [4/6]). During several outbreaks, two big-eyed pit vipers, two ball pythons, one ball python, one corn snake, and three cobras derived from colonies A–E, respectively, were submitted for postmortem examination at private laboratories. FFPE tissues (including lung, intestine, liver, spleen, kidney, and the male genital tract), plus additional fresh tissue samples derived from the necropsied snakes, were collected from private laboratories and used for studies at the Department of Pathology, Faculty of Veterinary Science, Chulalongkorn University. Information on necropsied samples used in this study is detailed in Table 1.

To explore the epidemiology of ferlavirus in Thai snakes, 252 samples comprised of oral (*n* = 126) and cloacal (*n* = 126) swabs were randomly collected from 126 individual pet snakes that were either healthy (*n* = 77) or diseased (*n* = 49). The samples were collected by inserting sterile rayon-tipped cotton swabs (Puritan; Guilford, ME, USA) into the oral cavity and cloacal canal of collected snakes. The swabs were then immersed in 1% phosphate buffer saline (PBS) and kept at –80 °C until assayed. The study design and protocol were approved by the Chulalongkorn University Animal Care and Use Committee (No. 1931036). All procedures were performed in accordance with relevant guidelines and regulations. Authors confirm that this study is reported in accordance with ARRIVE guidelines.

### Nucleic acid extraction and RT-PCR

Available fresh tissues and swab samples were subjected to viral nucleic extraction. The fresh tissues were individually homogenized in 0.5% PBS solution, except for male genital tract samples that were pooled before homogenizing. The homogenized tissues were then centrifuged, and the supernatant collected and used for further nucleic acid isolation using a Viral Nucleic Acid Extraction Kit II (GeneAid, Taipei, Taiwan) according to the manufacturer’s recommended procedure. The extracted viral nucleic acids were then quantified and qualified using a spectrophotometer (Nanodrop Lite; Thermo Fisher Scientific Inc., Waltham, MA, USA). The nucleic acids were then kept at − 80 °C until molecular assays were conducted.

Extracted nucleic acids were subsequently subjected to RT-PCR for ferlavirus detection. The RT-PCR reactions were done using a one-step RT-PCR kit (Qiagen, Hilden, Germany), intermixed with two sets of broad-ranged pan-paramyxovirus-family primers (PAR^30^ and PMX primers^31^) with amplification steps as previously described^30,31^. The RT-PCR products were initially visualized and analyzed using high throughput capillary electrophoresis with cartridge and protocol settings as previously described settings ^32^. Purified canine distemper virus (CDV) RNA^33^ and non-template samples served as positive and negative controls, respectively. Samples that presented target amplicons of both RT-PCR reactions were considered positive for paramyxovirus detection. Target amplicons were electrophoretically isolated using 1.5% agarose gel electrophoresis in 0.5 × Tris–borate-EDTA (TBE), subsequently purified using NucleoSpin Extract II (Macherey–Nagel, Düren, Germany) following the manufacturer’s protocols, and bidirectionally sequenced (Macrogen Inc., Seoul, South Korea) for additional confirmation.

Furthermore, all extracted viral nucleic samples were further processed for detection of other viruses using selected pan-virologic family RT-PCRs targeting reptilian reoviruses, arenaviruses, and retroviruses to elucidate other potential co-infections. Primers and protocols for these virus detections were utilized as previously described^34–37^.

### Whole genome characterization and phylogenetic analysis

Four pan-paramyxovirus RT-PCR-positive samples, including two from pooled male genital tracts (from colonies A and E), one lung (from colony B), and one liver (from colony D), were selected for whole genome sequencing and characterization. Multiple primer sets that are specific to snake ferlaviruses were designed based on the alignments of previously published ferlavirus sequences available in GenBank. The primers used for whole genome characterization and sequencing are described in Supplementary Table S1. Multiple RT-PCR assays were performed with various optimum annealing temperatures according to the annealing temperature (Ta) of each primer pair (in accordance with using the RT-PCR kit as described above). The positive target amplicons were isolated, purified, and sequenced using the protocols described above. The obtained sequences were initially allegorized to other available ferlavirus sequences deposited in GenBank using the nucleotide BLAST (BLASTn) algorithm. The obtained sequences were aligned using the MAFFT algorithm v. 7. Subsequently, whole genomes were constructed using BioEdit v. 7.2. (Ibis Biosciences, Carlsbad, CA, USA). Multiple series of transversional models, in proportion to invariable sites, and substitution models, according to the Bayesian information criterion (BIC) embedded in MEGA 7, were tested to find the best-fit model for the phylogenetic tree construction. The full-length ferlavirus sequence alignments were then used to construct the phylogenetic tree using maximum likelihood (ML) methods, which was performed with 1,000 bootstrap replicates. The pairwise nucleotide distances of detected ferlavirus complete genomes were calculated using BioEdit v.7.2.

### Histology and tissue localization of ferlavirus

The FFPE sections, derived from various private laboratories responsible for postmortem investigation of diseased snakes, were gathered and further processed histologically with routine staining. Initially, the slides were examined by two Thai board-certified veterinary pathologists (AR, WB) and then by one American board-certified veterinary pathologist (TK). Information regarding pathological descriptions was collected for interpretation.

To confirm the ferlavirus infection and to elucidate the association of displayed tissue pathology where ferlaviral genomes were presented, the ISH technique was performed. A ferlavirus probe, 600 bp and covering the partial polymerase gene (L), was constructed using a PCR DIG Probe Synthesis Kit (Roche Diagnostics, Basel, Switzerland) according to the manufacturer’s protocols. The ferlaviral-DIG (digoxigenin) probe was constructed under the same thermal cycling conditions described earlier using the pan-paramyxovirus RT-PCR primers with the additional use of DIG (DIG-labeled oligonucleotides) instead of the normal oligonucleotides. The constructed hybridization probe was validated by visualizing its size on 1.5% (w/v) agarose gel electrophoresis and by using control DNA as recommended by the manufacturer. The ISH procedure with a chromogenic probe was performed as previously described with some modifications^38,39^. Briefly, after deparaffinization and rehydration, the slides were incubated with 0.2 N hydrochloric acid (HCl) at room temperature for 20 min, followed by incubating in citrate buffer (pH6) at 95 °C for 20 min, and then treating with 10 ng/mL proteinase K (VWR, Radnor, PA, USA) at 37 °C for 20 min. Slides were then post-fixed with 0.4% formaldehyde solution. Thereafter, slides were prehybridized in prehybridization buffer (50% [v/v] formamide in 4X saline-sodium citrate [SSC] buffer), and subsequently treated overnight in an automated slide incubator with a hybridization buffer containing 20X SSC, 5X Denhardt’s solution, 100 µg/mL salmon sperm DNA, 0.5% (w/v) sodium dodecyl sulfate, and 10 ng of ferlavirus-DIG-labeled probe per slide at 50° C. Ferlavirus RT-PCR-positive tissue sections derived from a snake in colony A, incubated with a hybridization buffer containing DIG-labeled feline bocavirus-1 probe^38^, and the tissue section derived from ferlavirus RT-PCR-negative snake incubation with the constructed ferlavirus probe, served as negative controls. After overnight incubation, the slides were soaked in a series of gradient SCC buffers (2X SSC at 37 °C for 15 min, 1X SSC at 42 °C for 15 min, and 0.5X SSC at 42 °C for 15 min). The slides were subsequently incubated with blocking solution containing 5% bovine serum albumin (BSA) at room temperature for 30 min. After this non-specific blocking, the slides were incubated with 1:200 anti-DIG-AP Fab fragments (Roche, Basel, Switzerland) in 1X blocking solution. After multiple washing steps, hybridization signal detection was accomplished using liquid permanent red (LPR) (Dako, Glostrup, Denmark) applied in a dark chamber at room temperature for 20 min. Slides were then counterstained with hematoxylin prior to examination.

### Ultrastructural demonstration of ferlavirus particles

To demonstrate the ultrastructural localization and to confirm the results of hybridization of ferlavirus infection in the male reproductive tract of infected male snakes, FFPE sections of the vas deferens and epididymis, derived from two infected snakes (big-eyed pit viper No. 1 from colony A and cobra No. 1 from colony E), were subjected to TEM with a modified pop-off technique as previously described^40,41^. The TEM samples were prepared and double stained as previously published^40,41^. The sections were then examined using TEM (HT7800; Hitachi, Tokyo, Japan) operated at 80 kV.

### Ethics declarations

The authors confirm that the ethical policies of the journal, as noted on the journal’s author guidelines page, have been adhere to. This study was approved by Chulalongkorn University Animal Care and Use Committee (No. 1931036). All procedures were performed in accordance with relevant guidelines and regulations. Authors confirm that this study is reported in accordance with ARRIVE guidelines.

## Supplementary Information

Supplementary Information.

## Data Availability

The data that support the findings of this study are available in this manuscript. Four full-length coding sequences of obtained reptilian ferlavirus were submitted to the NCBI databases under the GenBank Accession Nos. MW976960-MW976963.
