# Supplementary material for: Epizootic reptilian ferlavirus infection in individual and multiple snake colonies with additional evidence of the virus in the male genital tract
Source: Sci Rep. 2021 Jun 16;11:12731. doi: 10.1038/s41598-021-92156-5 (PMC8208991; doi:10.1038/s41598-021-92156-5)
Supplement: Supplementary file 1 — Supplementary Information. [file 41598_2021_92156_MOESM1_ESM.pdf]

**Epizootic reptilian ferlavirus infection in individuals and multiple snake colonies with additional evidence of the virus in male genital tract**

**Chutchai Piewbang[1,2], Sabrina Wahyu Wardhani[2,3], Panida Poonsin[1,2], Poowadon Chai-in[4], Sitthichok Lacharoje[1], Thanyarat Saengdet[5], Taksa Vasaruchapong[6], Suwimon Boonrungsiman[4], Piyaporn Kongmakee[7], Wijit Banlunara[1], Anudep Rungsipipat[1], Tanit Kasantikul[8], Somporn Techangamsuwan[1,2,*]**

**Supplementary Table S1.** Primer sets used for whole genome characterization of reptilian ferlavirus

| Direction | Primer name | Primer sequences (5'-3') |
|---|---|---|
| **Forward** | FerP1_44F | TAAGTTGAAATTAGGATCA |
| **Reverse** | FerP1_1381R | GACATGTTTGGCTC |
| **Forward** | FerP2_912F | TATGGTATAAACACACGCTC |
| **Reverse** | FerP2_1748R | CACCAGTCTCCACATTGAT |
| **Forward** | FerP3_1728F | CAATCAATGTGGAGACTG |
| **Reverse** | FerP3_2836R | GGTAATACGGGAACACA |
| **Forward** | FerP4_2789F | ATATTTGAGGTYTGGGA |
| **Reverse** | FerP4_4137R | CCTGTGATGAYACTTTCA |
| **Forward** | FerP5_3960F | GAAACACCAGATGCAC |
| **Reverse** | FerP5_5128R | TCAAACATRACCCCTA |
| **Forward** | FerP6_4964F | TAG GAG CAA AGY TCTT |
| **Reverse** | FerP6_5962R | TGRGTTTGCCATTC |
| **Forward** | FerP7_5854F | TGATCCAGATYGAGATG |
| **Reverse** | FerP7_7367R | AGTGAGAATGTDGGTAT |

| | | |
|---|---|---|
| **Forward** | FerP8o_7532F | AAATCCTGCAGTACCGTGGCA |
| **Reverse** | FerP8o_7532R | AGATATCTGTGTAAACTCCTG |
| **Forward** | FerP8i_7647F | GCACTCTACCCCAGTGTTGG |
| **Reverse** | FerP8i_8045R | TTCTATCCAGCTTATTCTTAT |
| **Forward** | FerP1_44F | GCAGAGATTTTCTCTTTCTT |
| **Reverse** | FerP1_1381R | AGCTCTCATTTTGTATGTCAT |
| **Forward** | FerP9o_9618 | TAGAGGCTGTTACTGCTGC |
| **Reverse** | FerP9o_10244 | CATCTTGGCAAATAATCTGCC |
| **Forward** | FerP9i_9661F | ATGAGAGCYGCACARGT |
| **Reverse** | FerP9i_10226R | ACTGCRGAGACTCTCAC |
| **Forward** | FerP12_10393F | GTGAGAGTCTCYGCAGT |
| **Reverse** | FerP12_11024R | CTRGCACCTGTCACRGA |
| **Forward** | FerP13_11008F | TAGACTGGGCATCRGATCC |
| **Reverse** | FerP13_11933R | CCRAGBACCCAATATCTRCT |
| **Forward** | FerP14_11693F | AARTGGACAATTTCHGG |
| **Reverse** | FerP14_13691R | TCTGAGTGAACRARTGA |
| **Forward** | FerP15_13453F | CYTGGGTAGGRAATGA |
| **Reverse** | FerP15_14384R | AGGGRAAATATGAAYAAG A |
| **Forward** | FerP16_14306F | ATCCAATAAGAATAAGCTGGAT |
| **Reverse** | FerP16_15376R | GACCAAAGGATCGGAA |
| **Forward** | FerP17_8094F | ACTCAACCGGGTGTGTTAG |
| **Reverse** | FerP17_9801R | GTATGGTTTCATCAGTCCTCCAT |
| **Forward** | FerP18_7329F | GCAGAGATTTTCTCTTTCTT |
| **Reverse** | FerP18_7804R | AGCTCTCATTTTGTATGTCAT |

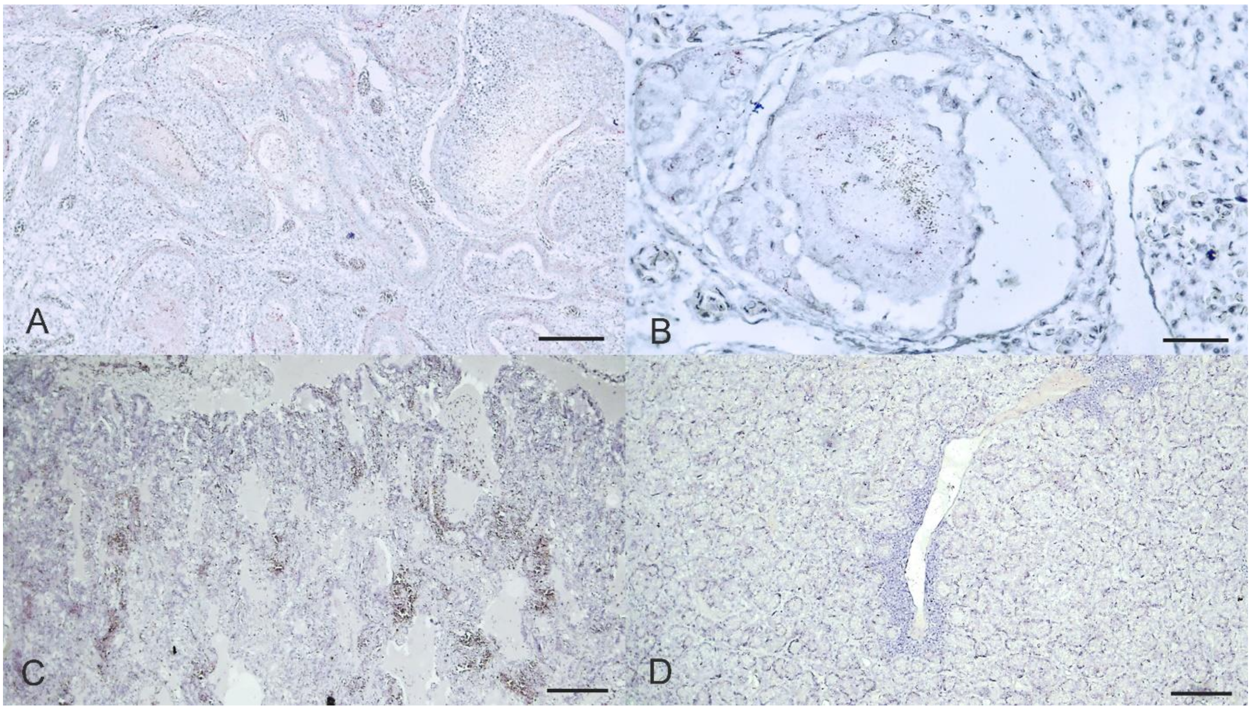


**Supplementary Fig. S1.** Reptilian ferlavirus infection. *In situ* hybridization. Epididymis. (A &B). Big-eyed viper (colony A). No hybridization signals were detected in the sections incubating with feline bocavirus probe. Lung (C.) and kidney(D). Boa constrictor (ferlavirus-PCR negative sections). No hybridization signals were found in the ferlavirus-PCR negative sections incubating with ferlavirus ISH probe. Bars indicate 250 µm.
